# Functional analysis of two mutation sites in the *OCA2* gene

**DOI:** 10.1038/s41598-024-64782-2

**Published:** 2024-06-26

**Authors:** XiaoHua Yuan, Qun Dang, Xue Lan Li

**Affiliations:** 1 Department of Gynaecology and Obstetrics, The First Affiliated Hospital of Xi’an Jiaotong University, No. 277, Yanta West Road, Xi’an, 710061 China; 2 Department of Gynaecology and Obstetrics, Shaanxi Provincial People’s Hospital, Xi’an, 710068 China

**Keywords:** *OCA*2 gene, Expression, Truncated analysis, Truncated protein, Oculocutaneous albinism, Mutation, Mechanisms of disease

## Abstract

To analyse the genetic aetiology of a child with oculocutaneous albinism and to explore the effects of two mutation sites on the function of the OCA2 protein at the mRNA and protein levels via the use of recombinant carriers in vitro. Whole-exome sequencing (WES) and Sanger sequencing were used to analyse the pathogenic genes of the child and validate the mutations in the parents. pEGFP and phage vectors carrying wild-type and mutant *OCA2* were constructed using the coding DNA sequence (CDS) of the whole gene-synthesized *OCA2* as a template and transfected into HEK293T cells, after which expression analysis was performed. The child in this study was born with white skin, hair, eyelashes, and eyebrows and exhibited nystagmus. Genetic analysis indicated that the child carried two heterozygous mutations: c.1079C > T (p.Ser360Phe) of maternal origin and c.1095_1103delAGCACTGGC (p.Ala366_Ala368del) of paternal origin, conforming to an autosomal recessive inheritance pattern. In vitro analysis showed that the expression of the c.1079C > T (p.Ser360Phe) mutant did not significantly change at the mRNA level but did increase at the protein level, suggesting that the mutation may lead to enhanced protein stability, and the c.1095_1103delAGCACTGGC (p.Ala366_Ala368del) mutation resulted in the loss of three amino acids in exon 10, producing a truncated protein. In vitro expression analysis also revealed that the expression of the mutant gene was significantly downregulated at both the mRNA and protein levels, suggesting that the mutation can simultaneously produce truncated proteins and lead to protein degradation. This case study enriches the phenotypic spectrum of *OCA2* gene disease. In vitro expression analysis confirmed that both mutations affect protein expression, providing a theoretical basis for analysing the pathogenicity of these two mutations.

## Introduction

Albinism, also known as oculocutaneous albinism (OCA), generalized albinism, leukopathia, and congenital achroma, is an autosomal recessive skin disease manifested as partial or complete loss of pigmentation of the skin, hair, and eyes. OCA can be divided into nonsyndromic OCA and syndromic OCA according to whether systems or organs other than the skin, hair, or eyes are involved. OCA has an incidence rate of 1:17,000 and a carrier rate of 1/70 in the human population. The incidence rate of OCA is as high as 1:1000 in some African countries due to ethnocultural and consanguineous marriages and 1:18,000 in the Chinese population^1^. OCA is genetically heterogeneous, and a variety of genetic mutations associated with melanin formation and transport can lead to the disease phenotype. The *TYR*, *OCA2*, *TYRP1*, and *SLC45A2* genes cause OCA types 1–4; *SLC24A5* and *LRMDA* cause OCA types 6–7; and the *OCA5* mutation is localized to the chromosome 4q24 region^2^. In addition, mutations in the *GPR143* gene result in an X-chromosome-linked albinism phenotype limited to the eye, also known as ocular albinism (OA1). Albinism type 2 is caused by mutations in the *OCA2* gene, and these mutations are the second largest class of albinism-causing genetic mutations in the Chinese population^1^. In this study, we reported two compound heterozygous mutations in the *OCA2* gene in an albino child and elucidated their effects on protein function by in vitro expression and bioinformatics analyses.

## Study subject

An 8-year-old female child was admitted to the hospital with the chief complaint of "high myopia in both eyes for more than 3 years." She was the second child (G2P1) and delivered by caesarean section at full term, with a birth weight of 2.78 kg. Any similar family history of hereditary disease was denied. The child presented with white skin, body hair, eyelashes, and eyebrows; nystagmus; and high myopic choroidal retinopathy in both eyes. Both eyes had clear corneas, normal anterior chamber depth, clear aqueous humour, round pupils, and bluish-white irises lacking melanin that were approximately 3 mm in diameter. Additionally, the eyes were found to exhibit light reflexes, but the lens was clear with fundus albinos and high myopia fundus changes.

## Methods

### Sample collection and genetic analysis

All methods were performed in accordance with the relevant guidelines and regulations. This study was approved by the child’s guardian and the ethics committee of The First Affiliated Hospital, Xi'an Jiaotong University. After the parents of the child signed the informed consent for genetic testing, 3 ml of peripheral blood from the child and her parents were drawn using EDTA anticoagulation tubes. The whole-genome DNA of the child and her parents was extracted using a QIAamp DNA Blood Mini Kit according to the manufacturer’s instructions for detection of the entire exome of the family.

One microgram of genomic DNA was utilized to construct a whole-genome library through PCR-free technology after disruption, and whole-exome DNA hybridization was performed using a NanoWES probe and enriched for high-throughput sequencing (Illumina NovaSeq 6000). Afterwards, bioinformatics analysis was performed by Berry Genetics on the sequenced data, and the raw data were subjected to quality control processing and compared with the human reference genome hg38/GRCh38 (BWA, Burrows‒Wheeler Alignment). The data were analysed using the Verita Trekker® Mutation Site Detection System and the Enliven® Mutation Site Annotation and Interpretation System; mutation sites with a mutation frequency greater than 1% in the 1000G, gnomAD, dbSNP, and internal databases, as well as nonfunctional mutation sites (e.g., synonymous mutations, noncoding region mutations) were removed. Pathogenicity prediction was performed (SIFT, PolyPhen2, CADD, etc.) to identify the candidate mutation sites for lineage validation in combination with a comprehensive assessment of clinical symptoms, relevant disease databases, and references. The pathogenicity ratings of the mutation sites and the rules of data interpretation were based on the American College of Medical Genetics and Genomics (ACMG) guidelines and recommendations of the ClinGen Sequence Variant Interpretation (SVI) expert group^3^.

Sanger sequencing was used for lineage validation based on the candidate mutation sites screened by WES, and two pairs of specific primers were designed for Touch Down PCR amplification, with the following amplification conditions: 95 °C for 5 min; 10 cycles of 95 °C for 30 s, 60 °C for 30 s, and 72 °C for 30 s; 20 cycles of 95 °C for 30 s, 55 °C for 30 s; and 72 °C for 30 s; and extension at 72 °C for 5 min. PCR products were detected by 1% agarose gel electrophoresis, and Sanger sequencing was completed on an ABI 3500DX sequencer after confirming the target bands.

### Wild-type (wt) and mutant (mut) expression vector construction

#### pEGFP-C1-OCA2 vector construction

In this study, both pEGFP and phage expression vectors were constructed for experimental analysis to obtain more reliable experimental results.

1. pEGFP-C1-wt vector construction

The XhoI-wt-BamHI fragment was obtained after amplification using the whole-gene synthesized *OCA2* coding DNA sequence (CDS) as the template and pEGFP-C1-OCA2-XhoI-F/pEGFP-C1-OCA2-BamHI-R as the primers. After double cleavage by XhoI and BamHI, the pEGFP-C1-wt vector was generated to obtain the pEGFP-C1 vector for sequencing verification.

(2) pEGFP-C1-mut1 (c.1079C > T: p.Ser360Phe) vector construction

With the pEGFP-C1-wt vector as the template, the mut1-1 fragment was obtained with pEGFP-C1-OCA2-XhoI-F/OCA2-mut1-R as the PCR primers, and the mut1-2 fragment was obtained with OCA2-mut1-F/pEGFP-C1-OCA2-BamHI-R as the PCR primers. A 1:1 mixture of mut1-1 and mut1-2 comprised the template for a second round of PCR amplification using the primers pEGFP-C1-OCA2-XhoI-F and pEGFP-C1-OCA2-BamHI-R to obtain the XhoI-mut1-BamHI fragment. The pEGFP-C1-mut1 vector was obtained by double cleavage of the mut1 fragment and the pEGFP-C1-wt vector by XhoI and BamHI, followed by recovery and ligation.

(3) pEGFP-C1-mut2 (c.1095_1103delAGCACTGGC: p.Ala366_Ala368del) vector construction.

With the pEGFP-C1-wt vector as the template, the mut2-1 fragment was obtained with pEGFP-C1-OCA2-XhoI-F/OCA2-mut2-R as the PCR primers, and the mut2-2 fragment was obtained with OCA2-mut2-F/pEGFP-C1-OCA2-BamHI-R as the PCR primers. A 1:1 mixture of mut2-1 and mut2-2 was used as the template for a second round of PCR amplification employing the primers pEGFP-C1-OCA2-XhoI-F and pEGFP-C1-OCA2-BamHI-R to obtain the XhoI-mut2-BamHI fragment. The pEGFP-C1-mut2 vector was generated by double cleavage of the mut2 fragment and the pEGFP-C1-wt vector by XhoI and BamHI, followed by recovery and ligation.

#### phage-OCA2 vector construction

1. phage-wt vector construction

The SalI-wt-NotI fragment was obtained after amplification with the whole-gene synthesized *OCA2* CDS as the template and phage OCA2-SalI-F/phage OCA2-NotI-R as the primers. After double cleavage by SalI and NotI, the phage vector was generated to obtain the Phage-wt vector for sequencing verification.

(2) phage-mut1 (c.1079C > T: p.Ser360Phe) vector construction

With the phage-wt vector as the template, the mut1-1 fragment was obtained with phage-OCA2-SalI-F/OCA2-mut1-R as the PCR primers, and the mut1-2 fragment was obtained with OCA2-mut1-F/phage-OCA2-NotI-R as the PCR primers. A 1:1 mixture of mut1-1 and mut1-2 was used as the template for a second round of PCR amplification using phage-OCA2-SalI-F and phage-OCA2-NotI-R as primers to obtain the SalI-mut1-NotI fragment. The Phage-mut1 vector was obtained by double cleavage of the mut1 fragment and the phage-wt vector by SalI and NotI, followed by recovery and ligation.

(3) phage-mut2 (c.1095_1103delAGCACTGGC: p.Ala366_Ala368del) vector construction

With the phage-wt vector as the template, the mut2-1 fragment was obtained with phage-OCA2-SalI-F/OCA2-mut2-R as the PCR primers, and the mut2-2 fragment was acquired with OCA2-mut2-F/phage-OCA2-NotI-R as the PCR primers. A 1:1 mixture of mut2-1 and mut2-2 was used as the template for a second round of PCR amplification employing phage-OCA2-SalI-F and phage-OCA2-NotI-R as primers to obtain the SalI-mut2-NotI fragment. The phage-mut2 vector was generated by double cleavage of the mut2 fragment and the phage-wt vector by SalI and NotI, followed by recovery and ligation.

### PCR amplification

PCR amplification was performed using TaKaRa's PrimerSTAR MAX DNA Polymerase (R045A) in a 50 μl system at an annealing temperature of 57 °C for 30 cycles, after which agarose gel electrophoresis was used to detect the amplification products, and a conventional gel was used to recover the target DNA fragments. The sequences of all primers are shown in Table 1. Primer Sequences.

**Table 1:** Primer Sequences.

Gene name	Primer name	Primer sequence
Vector construction	OCA2-mut1-F	CGGCCATGCTGGGTTTCCTTGCAGCACTGG
	OCA2-mut1-R	CCAGTGCTGCAAGGAAACCCAGCATGGCCG
	OCA2-mut2-F	GGCCATGCTGGGTTCCCTTGCAGCACTGGCTGT GATTGGCGATAGACCCAGCCTGACCCA
	OCA2-mut2-R	TGGGTCAGGCTGGGTCTATCGCCAATCACAGCC AGTGCTGCAAGGGAACCCAGCATGGCC
	OCA2-mut2-bacterial test-R	CGCCAATCACAGCCAGTGCT
	pEGFP-C1-OCA2-XhoI-F	AGATCTCGAGacATGCATCTGGAGGGCAGAGA
	pEGFP-C1-OCA2-BamHI-R	CGGTGGATCCTTAATTCCATCCCACCACCA
	phage-OCA2-SalI-F	TGACGTCGACaATGCATCTGGAGGGCAGAGA
	phage-OCA2-NotI-R	CGACGCGGCCGCtATTCCATCCCACCACCACATG
QPCR	OCA2-phage-QPCR-F	GCTAGCCTGACGTCGACaAT
	OCA2-phage-QPCR-R	GCCACAAGTTCAGCGAGTC
	OCA2-GFP-QPCR-F	ACATGGTCCTGCTGGAGTTC
	OCA2-GFP-QPCR-R	GGCCACAAGTTCAGCGAGT

### Enzyme digestion and linkage

The appropriate amounts of DNA fragments and vector plasmid were subjected to double enzyme digestion. After digestion for 2 h at 37 °C, agarose gel electrophoresis was used for detection, and a conventional gel was used to recover the target bands. After enzyme digestion, the ligation reaction system was prepared according to the following table for ligation at 4 °C overnight.

### Transformation and recombinant clone verification

After removing the product of overnight ligation, the DH5α competent cells were transformed by the conventional thermal stimulation method, followed by random selection of numerous monoclonal colonies for identification after overnight incubation at 37 °C; the identification methods included colony/bacteria solution PCR and Sanger sequencing.

### Cell transfection

Next, 293T cells were cultured in DMEM supplemented with 10% foetal bovine serum, and the constructed wild-type and mutant eukaryotic recombinant expression vectors were transiently transfected into 293T cells using Lipofectamine 2000 according to the manufacturer’s instructions. The samples were collected 48 h after transfection and subjected to QPCR and western blot assays.

### Expression analysis

The total RNA of cell samples collected 48 h after transfection with wild-type or mutant eukaryotic recombinant expression vectors was routinely extracted using the TRIzol method, followed by cDNA synthesis after DNA digestion, with QPCR detecting the expression levels of the target genes in the wild-type and mutant genotypes.

The total protein from the cell precipitate collected 48 h after transfection with the wild-type and mutant eukaryotic recombinant expression vectors was extracted using RIPA lysis buffer, followed by protein denaturation after determination of the protein concentration with a BSA kit. Equal amounts of total protein were subjected to SDS‒PAGE, and the expression of the wild-type and mutant target proteins was detected by western blotting.

## Results

### Genetic testing results

Whole-exome sequencing (WES) indicated that the child carried heterozygous mutations of the *OCA2* gene (NM_000275.2), c.1079C > T (p.Ser360Phe) c.1095_1103delAGCACTGGC (p.Ala366_Ala368del). The Sanger sequencing confirmed the WES results, showing that the father carried the c.1095_1103delAGCACTGGC heterozygous mutation and that the mother carried the c.1079C > T heterozygous mutation, consistent with an autosomal recessive inheritance pattern.

The c.1079C > T (p.Ser360Phe) mutation, which has been reported to be associated with albinism in the literature^4^, was undetected in normal control populations in the ESP database, the 1,000 Genomes Project database, or the gnomAD database and has been predicted to have deleterious effects on genes or gene products by a variety of statistical methods, including conservative prediction and evolutionary prediction. The phenotype of the child in this study was highly consistent with the albino phenotype. Therefore, this mutation was defined as clinically unspecified according to the ACMG guidelines (PM2 + PP3 + PP4).

c.1095_1103delAGCACTGGC (p.Ala366_Ala368del) is also a known mutation^5^ that has not been detected in the ESP database, the 1,000 Genomes Project database, or the gnomAD database in normal control populations and results in a shortened protein due to an in-frame deletion in a nonrepeat region. The phenotype of the child in this study was highly consistent with the albino phenotype. Therefore, this mutation was defined as clinically unspecified according to the ACMG guidelines (PM2 + PM4 + PP4).

### Results of vector construction

**Figure 1:**
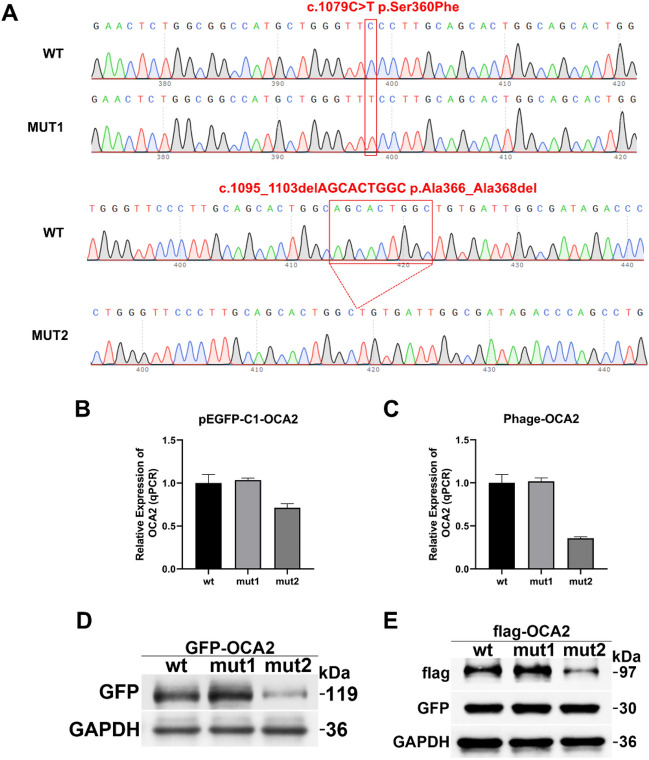
Effects of mutations on the expression of OCA2. (**A**) Chromatograms of c.1079C > T (p.Ser360Phe) and c.1095_1103delAGCACTGGC (p.Ala366_Ala368del) within GDAP2. The upper chromatograms represent normal sequences, and the lower chromatograms represent mutant sequences. (**B**,**C**) Relative expression of the *OCA2* mutant and wild-type mRNAs in the pEGFP-C1 (**B**) and phage (**C**) vectors. (**D**,**E**) Relative expression of the OCA2 mutant and wild-type proteins in the pEGFP-C1 (**D**) and phage (**E**) vectors. Anti-GFP antibodies were used as primary antibodies for the pEGFP-C1 vectors. Anti-FLAG antibodies were used as primary antibodies for the phage vectors.

Sanger sequencing revealed that both the wild-type and mutated plasmids were successfully constructed from both the GFP and phage vectors, and the sequencing results are shown in Fig. 1A.

### qPCR test results

The expression levels of the wild-type and mutant transcripts in the pEGFP-C1 and phage vectors were detected using the primers OCA2-GFP-QPCR-F/OCA2-GFP-QPCR-R and OCA2-phage-QPCR-F/OCA2-phage-QPCR-R, respectively.

In the pEGFP vectors, there was no significant change in the expression of the p.Ser360Phe missense mutation relative to the wild-type control, and the expression of the p.Ala366_Ala368del deletion mutation was reduced to 0.71 (Fig. 1B). In the phage vectors, there was no significant change in the expression of p.Ser360Phe relative to the wild-type control, and the expression of p.Ala366_Ala368del was reduced to 0.36 (Fig. 1C).

### Western blotting results

The expression levels of the wild-type and mut1/mut2 proteins in the pEGFP-C1 vector were detected using a GFP tag antibody. In the pEGFP-C1 vectors, the theoretical size of the wild-type protein was 119 kDa, the theoretical size of mut1 was 119 kDa, and the theoretical size of mut2 was 119 kDa. The western blotting results showed that the protein expression of the p.Ser360Phe missense mutation was increased compared with that of the wild-type protein, and the protein expression of the p.Ala366_Ala368del deletion mutation was significantly reduced compared with that of the wild-type protein (Fig. 1D).

The expression levels of the wt and mut1/mut2 proteins in the phage vectors were detected using a FLAG tag antibody. In the phage vectors, the theoretical size of the wild-type protein was 97 kDa, the theoretical size of the mut1 protein was 97 kDa, and the theoretical size of the mut2 protein was 97 kDa. Western blotting showed that the protein expression of the p.Ser360Phe missense mutation was increased compared with that of the wild-type protein, and the protein expression of the p.Ala366_Ala368del deletion mutation was significantly reduced compared with that of the wild-type protein (Fig. 1E).

### Protein 3D structure prediction

**Figure 2:**
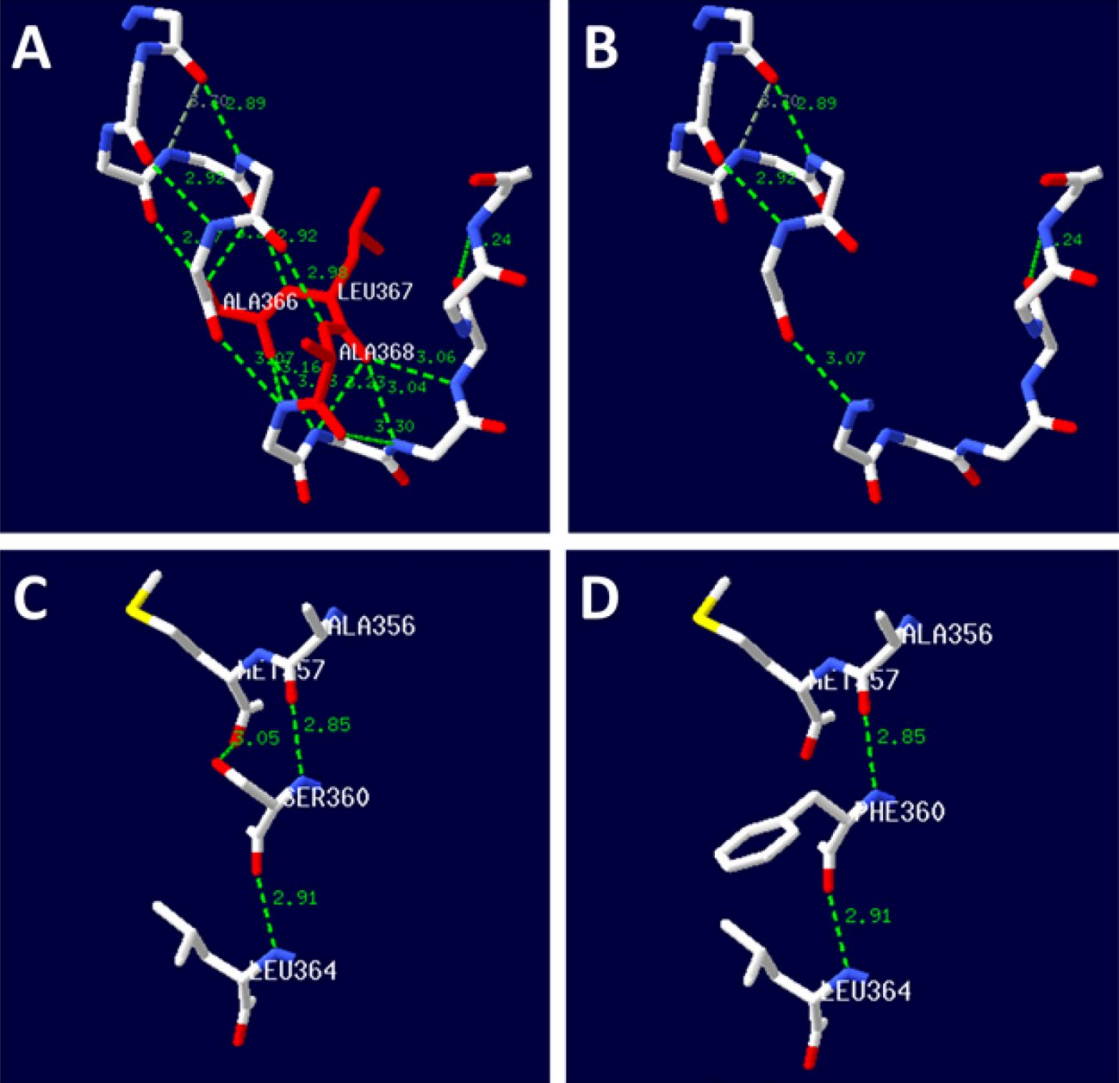
3D protein structure prediction of the OCA2 protein. Amino acid and conformation changes of the wild-type (**A**) and p.Ala366_Ala368del mutant (**B**) proteins. In the wild-type protein, ALA366, LEU367, ALA368 and other amino acids were linked by hydrogen bonds. In the mutant protein, the hydrogen bonds between amino acids disappeared. Amino acid and conformation changes of the wild-type (**C**) and p.Ser360Phe mutant (**D**) proteins. In the wild-type protein, Ser360, ALA356, MET357 and LEU367 were linked by hydrogen bonds. In the mutant protein, the hydrogen bonds between Phe360 and MET357 disappeared.

SWISS-MODEL was used to simulate the prominent amino acid and conformational changes in the affected polypeptide. Amino acid and conformation changes were found to occur between the wild-type (C) and p.Ser360Phe mutant (D) proteins. In the wild-type protein, Ser360, ALA356, MET357 and LEU367 were linked by hydrogen bonds. After the mutation, the hydrogen bonds between Phe360 and MET357 disappeared, affecting intermolecular forces and possibly affecting protein stability (Fig. 2C,D). Amino acid and conformation changes were observed between the wild-type (A) and p.Ala366_Ala368del mutant (B) proteins. In the wild type, ALA366, LEU367, ALA368 and other amino acids were linked by hydrogen bonds. After the mutation, the absence of hydrogen bonds between amino acids affected intermolecular forces and may have affected protein stability (Fig. 2A,B).

## Discussion

In this study, two rare mutations of the *OCA2* gene, c.1079C > T and c.1095_1103delAGCACTGGC, were detected in the peripheral blood of a child with ocular albinism. However, there is a lack of functional studies on these two mutations, which are reportedly associated with albinism. In this study, we analysed the effects of two mutations on OCA2 protein expression by constructing two expression vectors, pEGFP and phage, in vitro and found that the c.1079C > T (p.Ser360Phe) missense mutation increased OCA2 protein expression compared to that of the wild type, while the p.Ala366_Ala368del deletion mutation significantly reduced protein expression compared with that of the wild type, which confirms the effects of the two mutations on protein expression at the molecular level and lays the foundation for further studies on the molecular mechanism of the protein.

OCA2 has a highly variable clinical phenotype. Affected individuals may have some melanosis, usually resulting in a lighter colour than that of unaffected family members. In other words, their hair colour is usually not entirely white, they have mild to moderate hypopigmentation of the skin and iris, and they may have nevi and pigmented spots in exposed areas, with the size of the pigmented spots increasing progressively. In people of African descent, a mutation in the P gene can result in a light brown skin phenotype known as "brown OCA". Most patients with type II OCA acquire small amounts of pigmentation with age^6,7^. Patients with type II OCA also have characteristic visual abnormalities associated with albinism, including decreased visual acuity and nystagmus, which are usually less severe than those in patients with type I OCA^4^. The c.1079C > T (p.Ser360Phe) mutation in the *OCA2* gene carried by the child in this study was previously reported in a 1-month-old Chinese child (also carrying the c.1096_1104del mutation), who was only reported to present with white hair, fair skin, unknown colour of the iris of the eyes, and pigmented nevus in the groin, as well as other ocular abnormalities that were not described due to the young age of the child^4^. The c.1095_1103delAGCACTGGC (p.Ala366_Ala368del) mutation was detected in a French cohort of patients with albinism who also carried a heterozygous mutation of c.1481C > T (p.Ser494Phe), with the detailed clinical phenotype not described^5^. The clinical manifestations of this child included hypopigmentation of the skin and hair, pigmented nevus on the waist, and the significant manifestation of decreased vision, suggesting that the care and prevention of vision should be emphasized in the clinical management of these children. The genotype of the child was similar to that of patients previously reported in the literature, expanding the phenotypic spectrum of the disease and enriching the disease database of the Chinese population.

*OCA2*, which is located at chromosome position 15q11.2-q12, contains 23 coding exons encoding a 110 kDa transmembrane protein, also known as the P protein, with 12 transmembrane helices. The P protein is specifically expressed in mature (stage III–IV) melanin bodies^8^ and acts as an ion channel protein that converts acidic pH to neutral pH, enabling melanin synthesis by maintaining the neutral pH environment required for tyrosinase activity^9–12^. Reduced P protein expression can lead to a decrease in melanin synthesis^13^. The p.Ser360Phe missense mutation carried by the child in this study is located in the 3rd transmembrane helix of the P protein, and its mutation is predicted to increase the stability of the P protein according to I-Mutant2.0^4^. The in vitro overexpression analysis in this study showed that the expression of the mutant protein carrying p.Ser360Phe did not change significantly at the mRNA level and increased at the protein level compared to that of the wild-type protein, suggesting that the mutation may lead to enhanced protein stability. This increased stability would cause a slower rate of degradation, thereby affecting the pH homeostasis of the cellular environment, which in turn has an impact on the activity of tyrosinase and results in the disruption of melanin synthesis. The other P protein mutation, p.Ala366_Ala368del, carried by the child in this study was also located in the 3rd transmembrane helix, which resulted in the absence of three amino acids at positions 366–368, which may destroy the stability and specificity of the transmembrane region. The in vitro expression assay indicated that the expression of the mutant protein was significantly downregulated at both the mRNA and protein levels, suggesting that the mutation produces a truncated protein and leads to protein degradation. This result implies that reduced protein expression cannot properly regulate pH homeostasis in cells, leading to reduced tyrosinase activity and reduced melanin synthesis, which in turn leads to the albino phenotype. However, the exact molecular mechanism by which these two mutations affect melanin synthesis requires further investigation.

In conclusion, this study clarified the pathogenesis of this disease in children from a genetic perspective, enriching the phenotypic spectrum of the disease and providing a strong molecular basis for prenatal diagnosis of the next birth in families, which can aid in the precise management of the disease's therapeutic management and prognosis. In addition, expression analysis was carried out at the mRNA and protein levels in this study, which clarified the effects of the two P protein mutations on protein expression, thus providing a theoretical basis for the analysis of the pathogenicity of this mutant site and laying an experimental foundation for subsequent functional studies.

### Supplementary Information

Supplementary Information 1.

Supplementary Information 2.

Supplementary Information 3.

Supplementary Information 4.

Supplementary Information 5.

## Data Availability

We have deposited database and the relevant accession numbers is PRJNA1063736 and the database is SRA. Our data will be released at 2028-01-09.

## References

[1] Zhong Z (2019). Comprehensive analysis of spectral distribution of a large cohort of Chinese patients with non-syndromic oculocutaneous albinism facilitates genetic diagnosis. Pigment Cell Melanoma Res..

[2] Kausar T (2013). OCA5, a novel locus for non-syndromic oculocutaneous albinism, maps to chromosome 4q24. Clin. Genet..

[3] Richards S (2015). Standards and guidelines for the interpretation of sequence variants: A joint consensus recommendation of the American College of Medical Genetics and Genomics and the Association for Molecular Pathology. Genet. Med..

[4] Lin Y (2019). Mutational analysis of TYR, OCA2, and SLC45A2 genes in Chinese families with oculocutaneous albinism. Mol. Genet. Genomic Med..

[5] Lasseaux E (2018). Molecular characterization of a series of 990 index patients with albinism. Pigment Cell Melanoma Res..

[6] Kamaraj B, Purohit R (2014). Mutational analysis of oculocutaneous albinism: A compact review. Biomed. Res. Int..

[7] Kerr R (2000). Identification of P gene mutations in individuals with oculocutaneous albinism in sub-Saharan Africa. Hum. Mutat..

[8] Park S (2015). Unrevealing the role of P-protein on melanosome biology and structure, using siRNA-mediated down regulation of OCA2. Mol. Cell Biochem..

[9] Bellono NW (2014). An intracellular anion channel critical for pigmentation. Elife.

[10] Brilliant MH (2001). The mouse p (pink-eyed dilution) and human P genes, oculocutaneous albinism type 2 (OCA2), and melanosomal pH. Pigment Cell Res..

[11] Sturm RA, Frudakis TN (2004). Eye colour: Portals into pigmentation genes and ancestry. Trends Genet..

[12] Visser M, Kayser M, Palstra RJ (2012). HERC2 rs12913832 modulates human pigmentation by attenuating chromatin-loop formation between a long-range enhancer and the OCA2 promoter. Genome Res..

[13] Eiberg H (2008). Blue eye color in humans may be caused by a perfectly associated founder mutation in a regulatory element located within the HERC2 gene inhibiting OCA2 expression. Hum. Genet..

