# Supplementary figures and images for: Functional analysis of two mutation sites in the OCA2 gene
Source: Sci Rep. 2024 Jun 26;14:14789. doi: 10.1038/s41598-024-64782-2 (PMC11208167; doi:10.1038/s41598-024-64782-2)

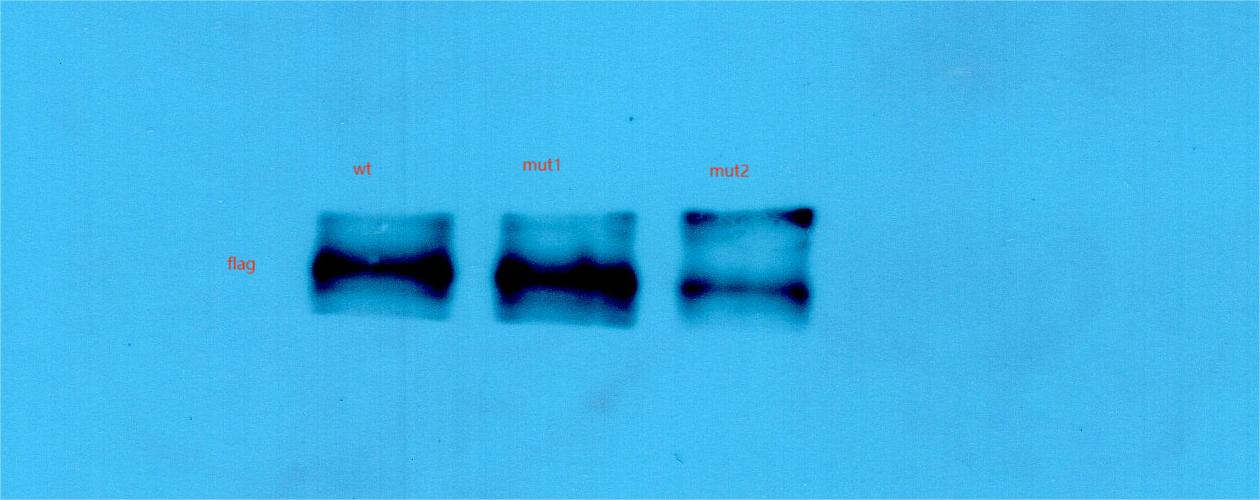

Supplement: Supplementary file 1 — Supplementary Information 1. [file 41598_2024_64782_MOESM1_ESM.jpg]

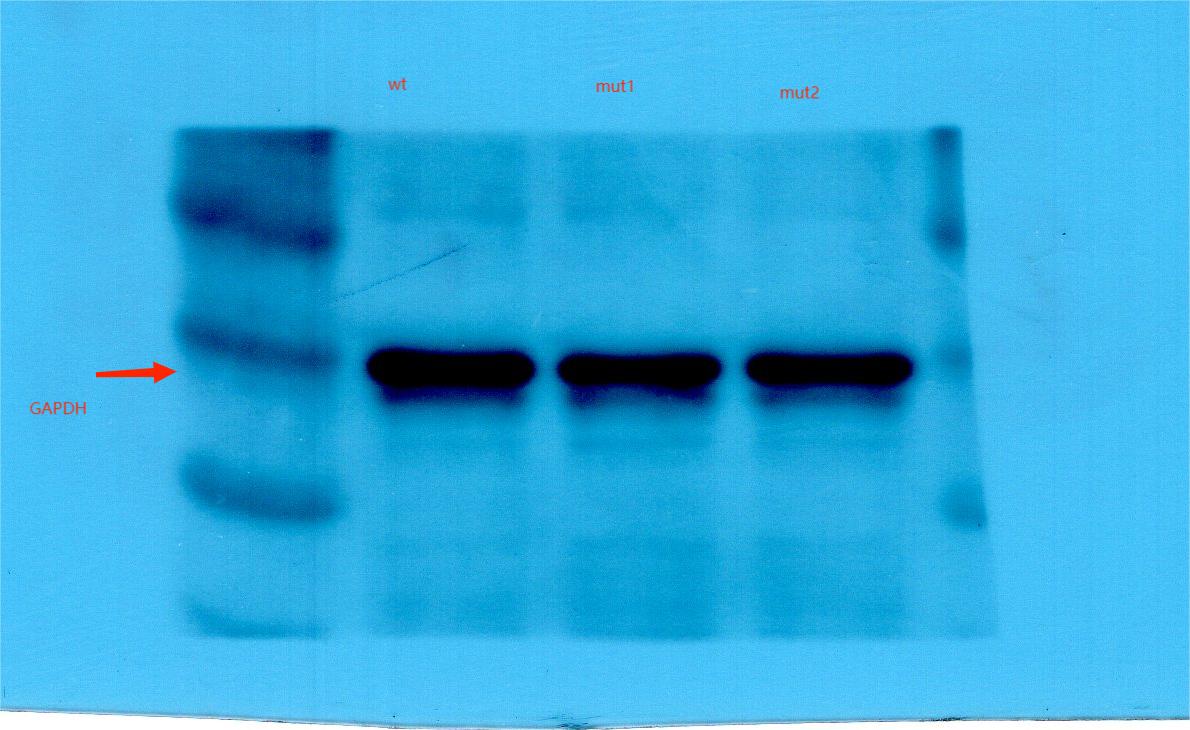

Supplement: Supplementary file 2 — Supplementary Information 2. [file 41598_2024_64782_MOESM2_ESM.jpg]

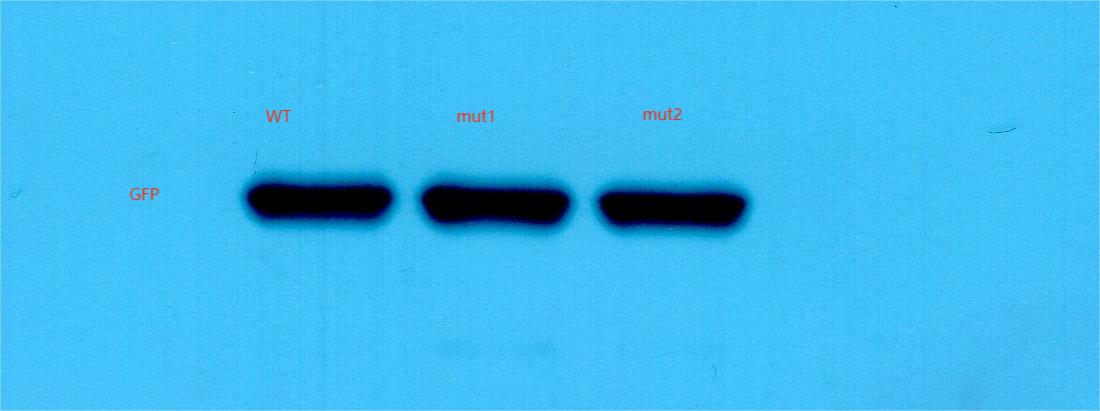

Supplement: Supplementary file 3 — Supplementary Information 3. [file 41598_2024_64782_MOESM3_ESM.jpg]

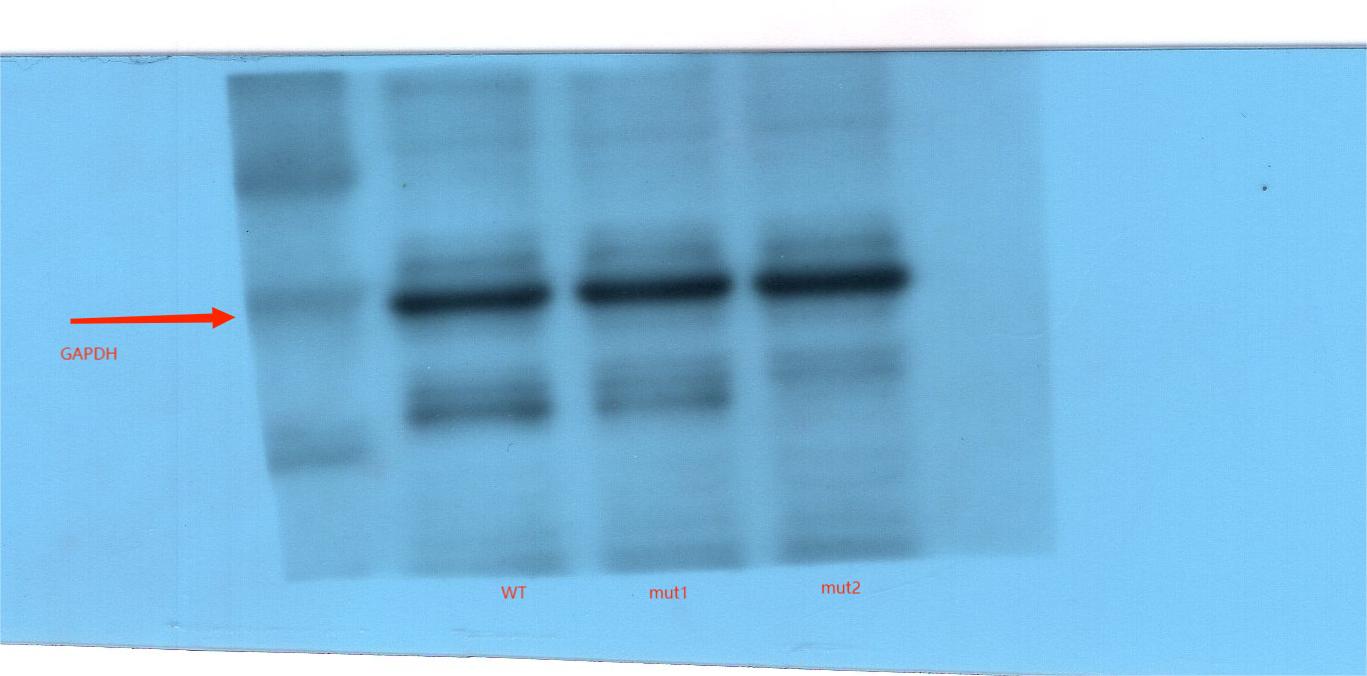

Supplement: Supplementary file 4 — Supplementary Information 4. [file 41598_2024_64782_MOESM4_ESM.jpg]

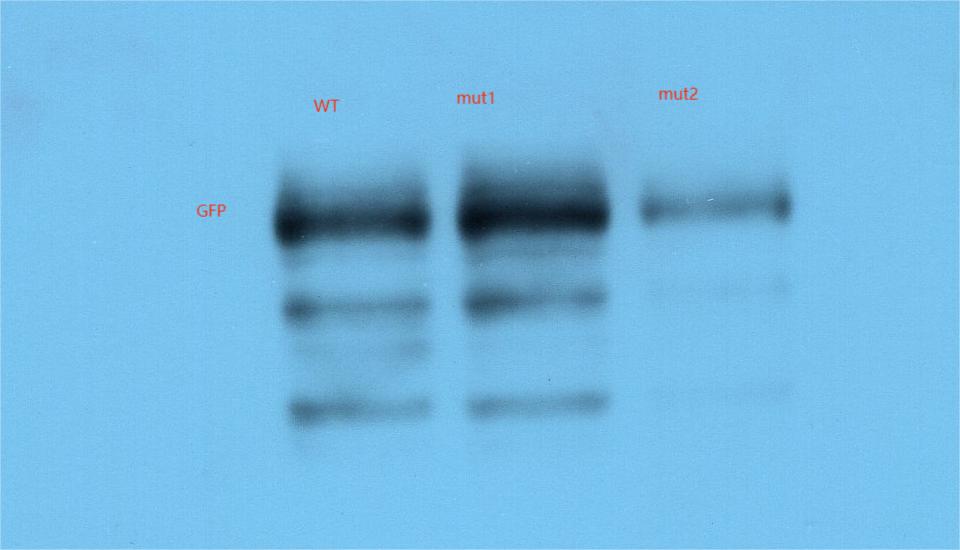

Supplement: Supplementary file 5 — Supplementary Information 5. [file 41598_2024_64782_MOESM5_ESM.jpg]
